# Functional connectivity of the sensorimotor cerebellum in autism: associations with sensory over-responsivity

**DOI:** 10.3389/fpsyt.2024.1337921

**Published:** 2024-03-25

**Authors:** Melis E. Cakar, Nana J. Okada, Kaitlin K. Cummings, Jiwon Jung, Susan Y. Bookheimer, Mirella Dapretto, Shulamite A. Green

**Affiliations:** ^1^Neuroscience Interdepartmental Program, University of California Los Angeles, Los Angeles, CA, United States; ^2^Department of Psychology, Harvard Medical School, Boston, MA, United States; ^3^Department of Psychiatry and Biobehavioral Sciences, University of California Los Angeles, Los Angeles, CA, United States; ^4^Jane and Terry Semel Institute for Neuroscience and Human Behavior, University of California Los Angeles, Los Angeles, CA, United States; ^5^Department of Psychology and Neuroscience, The University of North Carolina at Chapel Hill, Chapel Hill, NC, United States

**Keywords:** cerebellum, autism spectrum disorder, sensorimotor, sensory over-responsivity, functional connectivity, fMRI

## Abstract

The cerebellum has been consistently shown to be atypical in autism spectrum disorder (ASD). However, despite its known role in sensorimotor function, there is limited research on its association with sensory over-responsivity (SOR), a common and impairing feature of ASD. Thus, this study sought to examine functional connectivity of the sensorimotor cerebellum in ASD compared to typically developing (TD) youth and investigate whether cerebellar connectivity is associated with SOR. Resting-state functional connectivity of the sensorimotor cerebellum was examined in 54 ASD and 43 TD youth aged 8-18 years. Using a seed-based approach, connectivity of each sensorimotor cerebellar region (defined as lobules I-IV, V-VI and VIIIA&B) with the whole brain was examined in ASD compared to TD youth, and correlated with parent-reported SOR severity. Across all participants, the sensorimotor cerebellum was functionally connected with sensorimotor and visual regions, though the three seed regions showed distinct connectivity with limbic and higher-order sensory regions. ASD youth showed differences in connectivity including atypical connectivity within the cerebellum and increased connectivity with hippocampus and thalamus compared to TD youth. More severe SOR was associated with stronger connectivity with cortical regions involved in sensory and motor processes and weaker connectivity with cognitive and socio-emotional regions, particularly prefrontal cortex. These results suggest that atypical cerebellum function in ASD may play a role in sensory challenges in autism.

## 1 Introduction

Autism spectrum disorder (ASD) is a neurodevelopmental disorder characterized by socio-emotional and communicative difficulties, repetitive behaviors, and altered sensory processing (1). While the neurobiology underlying the etiology of ASD is quite complex, reflecting the considerable heterogeneity that characterizes this disorder, emerging research has consistently implicated cerebellar atypicalities in ASD (2–7). The cerebellum shows differences in structure, such as reduced Purkinje cell size and numbers, as well as function, such as atypical activation in the anterior cerebellum during motor tasks, in ASD (8–14). However, how these cerebellar atypicalities contribute to ASD features remains unclear. In particular, despite the recognized role of the cerebellum in sensorimotor processing (15), there are almost no studies that investigate the relationship between cerebellar function and sensory processing difficulties in ASD, which are known to be both extremely prevalent and a barrier to quality of life (16, 17).

More than 90% of children with ASD experience sensory processing atypicalities, and in particular at least 56-70% of youth with ASD experience sensory over-responsivity (SOR; 16, 18–20). SOR, characterized by an extreme discomfort in response to sensory stimulation, can be particularly impairing for children with ASD and has been previously linked to challenges with emotion regulation, communication, and daily-life adaptive skills (17, 21–23). Functional magnetic resonance imaging (fMRI) studies in the past decade have shown that SOR is associated with greater neural sensitivity and reduced neural habituation in sensory-limbic regions, such as the amygdala and primary sensory cortices during sensory stimulation (24, 25). Similar to other complex features of ASD, SOR involves multiple neural networks, including altered patterns in fronto-amygdala (24, 25), thalamus (26), and salience network connectivity (27, 28). Thus far, cerebellar circuits have not been investigated as a contributing factor to SOR in ASD. However, given the known role of the cerebellum in both sensorimotor processing and in ASD, the lack of cerebellum research in relation to SOR leaves a significant gap in our understanding of the neural mechanisms underlying this impairing feature of ASD.

While no studies to date have specifically investigated the link between the cerebellum and SOR, recent research has found evidence for atypical function of the cerebellum in sensorimotor processing in ASD. Specifically, a resting-state functional connectivity (rsFC) study has shown that the cerebellum is functionally *more* connected with cortical sensorimotor networks in youth with autism, suggesting a role for the cerebellum in atypical sensorimotor processing in ASD (11). Additionally, altered cerebellar functional connectivity was recently linked to broad sensory processing issues, as well as to socio-communicative signs of autism and restricted interests (29). Oldehinkel et al. (29) found that elevated connectivity between the cerebellum and somatosensory and motor cortices in ASD was associated with more severe sensory symptoms, further suggesting that atypicalities in the sensorimotor cerebellum may contribute to altered sensory processing in ASD. However, this particular study did not distinguish between different types of sensory processing symptoms in ASD, which vary widely and can, for example, include sensory *under*-responsivity and atypical sensation seeking in addition to SOR (30). Thus, the contribution of the cerebellum to specific sensory symptoms, particularly SOR (given its impairing nature) requires further study.

In the current study, we investigated differences in rsFC of the sensorimotor cerebellum in children and adolescents with ASD compared to typically developing peers (TD), as well as associations between cerebellar functional connectivity and SOR symptoms in ASD. We focused on the regions of the cerebellum that have previously been shown to play a key role in sensorimotor processing, namely lobules I-VI, VIIIA and VIIIB (9, 11, 15, 31–35). The anterior lobe of the cerebellum (lobules I-V and parts of VI) and lobules VIIIA and VIIIB are functionally connected with sensorimotor cortical regions (11, 36, 37) and house sensorimotor homonculi (15) – body maps analogous to that on the cerebral cortex. Within the anterior lobe, lobules V and VI have been reported to be particularly functionally connected with visual and auditory cortices (34, 36); accordingly, we distinguished lobules V-VI from lobules I-IV in our study. We also differentiated lobule VIII (i.e., lobules VIIIA and VIIIB) from lobules I-VI due to anatomical distance (i.e., anterior versus posterior cerebellum; 9). For each of the three sensorimotor cerebellar seed regions (lobules I-IV, V-VI, and VIII), we aimed to examine the differences in connectivity in ASD compared to TD youth, and the association between connectivity and SOR severity within youth with ASD.

## 2 Materials and methods

### 2.1 Participants

Participants were 54 ASD (16F) and 43 TD (10F) children and adolescents, aged 8.3 - 18.0 years. Written informed consent was obtained from all parents and from children who were 13 years or older; written assent was given by participants who were younger than 13 years. ASD diagnosis was confirmed with the Autism Diagnostic Interview-Revised (ADI-R; 38), Autism Diagnostic Observation Schedule - second edition (ADOS-2; 39), and best clinical judgment. Groups did not differ significantly in age, sex, ethnicity, race, or head motion (see **Table 1**). IQ was assessed using the Wechsler Abbreviated Scales of Intelligence (WASI; 40), and participants had a full-scale IQ of 75 or above. ASD participants had significantly lower full-scale IQ than TD participants (see **Table 1**), and thus, IQ was entered as a covariate in between-group analyses. Data were initially collected for 56 ASD and 47 TD participants. Participants with maximum absolute head motion (i.e., maximum head motion with respect to the reference volume) greater than 4mm and who were significant motion outliers for both maximum and mean absolute motion (i.e., mean head motion with respect to the reference volume) were excluded from our analysis (2 ASD and 1 TD). 3 TD participants were additionally excluded due to brain anomalies (2 TD) and elevated SOR severity (1 TD). All study procedures were approved by the University of California, Los Angeles, Institutional Review Board.

**Table 1:** Descriptives.

	*ASD (mean ± SD)*	*TD (mean ± SD)*	*t or χ^2^*
*N*	54	43	–
*Age (years)*	13.60 ± 2.95	13.02 ± 3.07	*p*=0.35
*Sex (females)*	16	10	*p*=0.48
*Ethnicity*			
*Hispanic or Latino/a*	18	13	*p*=0.83
*Not Hispanic or Latino/a*	36	30	
*Race*			
*American Indian/Alaska Native*	1	1	*p*=0.82^1^
*Asian*	6	9	
*Black or African American*	5	4	
*White*	33	23	
*More than One Race*	6	3	
*Unknown or Not Reported*	3	3	
*Scanner head motion*			
*Mean absolute motion^2^ (mm)*	0.40 ± 0.19	0.39 ± 0.19	*p*=0.82
*Mean relative motion^2^ (mm)*	0.13 ± 0.05	0.12 ± 0.04	*p*=0.35
*Components removed^3^*	111.87 ± 31.25	117.95 ± 30.80	*p*=0.34
*Components kept^3^*	142.81 ± 32.40	139.79 ± 27.25	*p*=0.62

*WASI full-scale IQ*	104.91 ± 16.21	114.44 ± 12.84	*p*=0.002
*SOR total score*	9.33 ± 7.47	1.05 ± 1.62	*p*<0.001
*Anxiety total score (SCARED Parent)*	18.06 ± 12.05	6.30 ± 6.54	*p*<0.001

^1^Fisher’s exact test was used to assess independence of the variables.

^2^Mean absolute motion and mean relative motion are values that refer to average head motion relative to the reference volume and previous volume, respectively. Both values were estimated using FSL’s MCFLIRT tool.

^3^Single-subject components that were identified by ICA-AROMA as motion or noise were regressed out (i.e., Components removed). The number of remaining components were reported as ‘Components kept.’

ASD, Autism spectrum disorder; TD, Typically developing; WASI, Wechsler Abbreviated Scale Intelligence; SOR, sensory over-responsivity; SCARED, Screen for Child Anxiety Related Disorders. Higher scores of SOR and SCARED indicate more severe SOR and anxiety, respectively.

### 2.2 Measures

Sensory Processing 3-Dimensions Scale (SP3D) Inventory (41) was used to assess SOR where parents indicated sensory experiences listed on the SP3D checklist that bother their child. The number of items (auditory, tactile or visual) were summed to calculate a total SOR score as in previous research (e.g. 42, 43), with a higher score denoting more severe SOR. Anxiety was measured using parent report on the Screen for Child Anxiety Related Emotional Disorder (i.e., SCARED) questionnaire (44) and included as a covariate in SOR analyses due to the high correlation between anxiety and SOR symptoms (e.g., 45).

### 2.3 MRI data acquisition

fMRI data were acquired on a Siemens Prisma 3-Tesla scanner with a 64-channel head coil. During the resting-state scan, participants fixed their gaze on a white crosshair on a black background, displayed using a pair of 800x640 resolution magnet-compatible 3D goggles under computer control (Resonance Technologies, Inc.). The resting-state scan was the first functional scan completed as part of a larger protocol. Scans were acquired using an EPI multiband acquisition lasting 8 minutes and covering the entire cerebral volume (TR=720ms, FOV=208 mm, TE=37ms, flip angle=52°, in-plane voxel size=2mm², 72 slices, multi-band acceleration factor=8).

### 2.4 Data preprocessing and analysis

#### 2.4.1 Behavioral data analysis

Group differences in SOR and anxiety were investigated with independent-samples t-tests using R Version 4.1.2. Because SOR is commonly reported as being correlated with anxiety (e.g., 45), we also examined the association between SOR and anxiety in the current study sample.

#### 2.4.2 Neuroimaging data analysis

The fMRI data were analyzed using the FMRIB Software Library (FSL)^1^, Version 5.0.11. Preprocessing steps included spatial smoothing (Gaussian kernel full width at half maximum=5mm) and the regression of mean white matter, cerebrospinal fluid, and global signal times series. To remove potential confounds resulting from head motion, Independent Component Analysis - Automatic Removal of Motion Artifacts (ICA-AROMA) was used, and single-subject components identified as motion or noise were regressed out (46, 47). Single-subject functional data were then registered to the MNI152 T1 2-mm template brain (12 degrees of freedom) using the registration matrix estimated prior to spatial smoothing.

We used seed-based connectivity analyses to examine functional connectivity between three regions of the sensorimotor cerebellum and the whole brain. Lobules I-IV, lobules V-VI and lobule VIII (including VIIIA and VIIIB) were defined as the three seed regions due to their association with sensorimotor processing (e.g., 9, 11, 15, 34). Cerebellar seeds were created with the Spatially Unbiased Infra-tentorial Template (SUIT; 48) probabilistic cerebellar atlas and thresholded at 75%, keeping only voxels with at least 0.75 probability of belonging to a certain lobule (i.e., belonging to a certain lobule in at least 75% of individuals included in the probabilistic atlas; **Supplementary Figure 1**). The 75% threshold was chosen for most complete lobule representation with the least overlap among lobules.

FSL’s fMRI Expert Analysis Tool (FEAT, version 6.00) was utilized to run a fixed-effects model for each subject, and FSL’s Local Analysis of Mixed Effects State (FLAME 1 + 2; 49–51) was used for higher-level group analyses. For each cerebellar seed, the time-series from the cerebellar region were isolated in individual subject space and correlated with neural activity in every other voxel in the brain to create single-subject connectivity maps. Prior to performing group level analyses, z-statistic maps were generated using Fischer’s r-to-z transformation. All whole-brain contrasts were corrected for multiple comparisons using Gaussian random-field theory (i.e., a type of Family-Wise Error (FWE) rate correction) in FSL with a voxel-wise threshold of *z*>3.1 (within-group contrasts) or *z*>2.3 (between-group contrasts and correlations with SOR) and a cluster-corrected threshold of *p*<0.05. Supplemental between-group and SOR correlation analyses were conducted at a more stringent voxel-wise threshold of z>2.7 and included in the **Supplementary Information (Supplementary Figures 2** and **3)**.

To assess SOR correlations with cerebellar rsFC in ASD, SOR was entered as a bottom-up regressor in each whole-brain analysis (one for each of the three cerebellar seed regions). Anxiety severity was included as a covariate in these analyses to examine the unique effect of SOR over and above anxiety, due to previously reported high correlations between SOR and anxiety symptoms (e.g., 45, 52). Parameter estimates indexing connectivity strength were extracted from each cluster showing a significant correlation with SOR and plotted to identify potential outliers. Supplemental analyses were conducted to assess the effect of anxiety on cerebellar connectivity over and above the effect of SOR severity (see **Supplementary Information**).

All analyses (i.e., within-group, between-group and SOR correlations) were repeated with age as a covariate of no interest to ensure that our findings were not due to age variability in the sample (see **Supplementary Information**).

## 3 Results

### 3.1 Behavioral measures

To examine group differences in SOR and anxiety, we performed independent-samples t-tests. The ASD group had significantly higher SOR [*t* (59.17)=7.92, *p*<0.001, 95% confidence interval= (6.19, 10.38); **Table 1**) and anxiety symptoms [*t* (84.82)=6.12, *p*<0.001, 95% confidence interval= (7.94, 15.57)] compared to the TD group. SOR was positively correlated with anxiety in the ASD sample (*r*=0.32, *p*=0.02), but not in the TD sample (*r*=0.06, *p*=0.71).

### 3.2 Cerebellar connectivity

**Figure 1:**
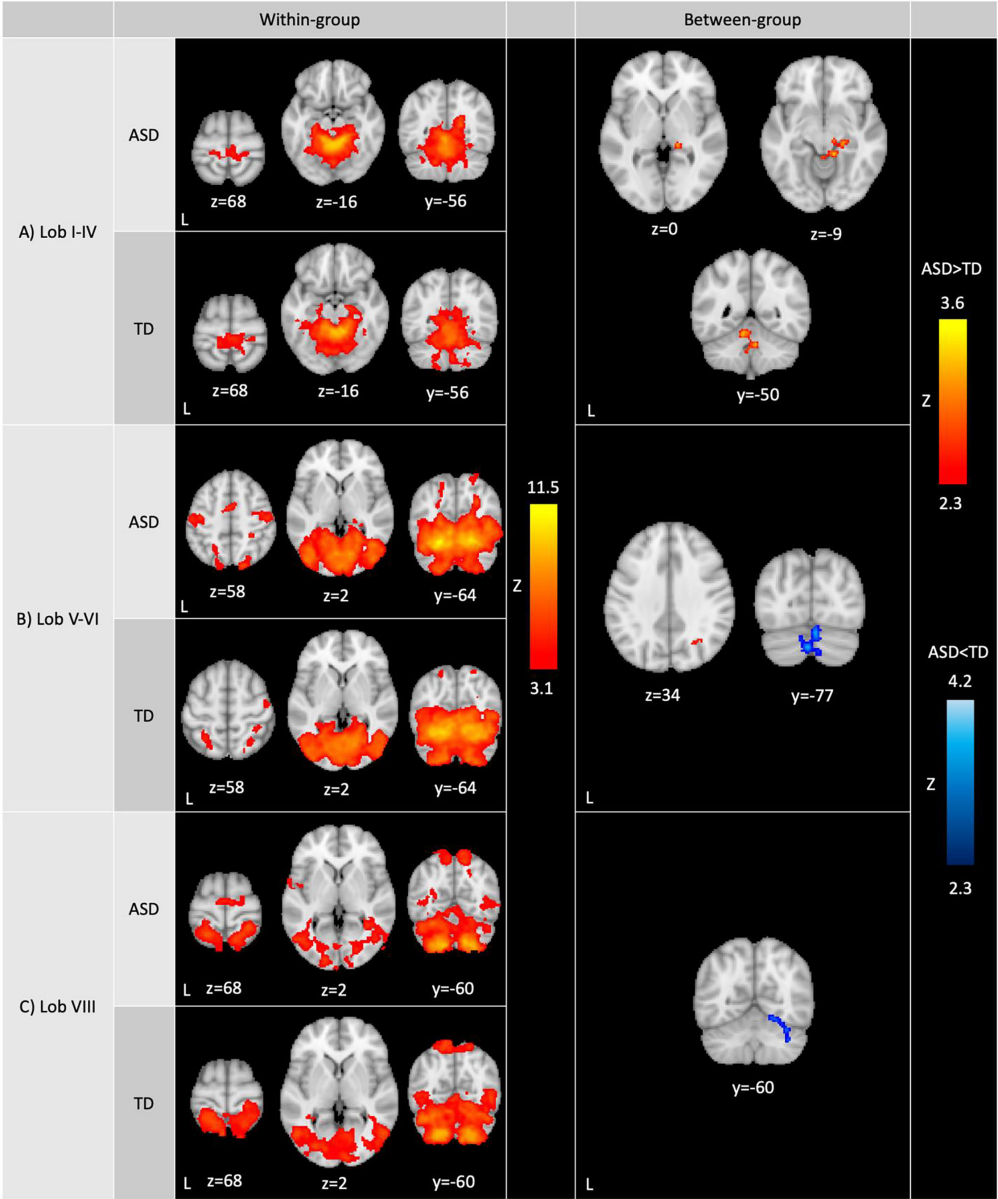
Whole-brain resting-state functional connectivity of the sensorimotor cerebellum. Within-group (left): Within-group ASD and TD functional contrasts were thresholded at Z>3.1, cluster corrected at p<0.05. Between-group (right): Between-group functional contrasts were thresholded at Z>2.3, cluster corrected at p<0.05. Full-scale IQ was included as a covariate in between-group analyses. Cerebellar lobules showed differences in connectivity between ASD and TD groups (red: ASD>TD; blue: ASD<TD). ASD>TD was masked by the ASD within-group contrast to display clusters that show greater positive connectivity in ASD compared to TD. Similarly, ASD<TD was masked by the TD within-group contrast to display clusters that show reduced positive connectivity in ASD compared to TD. ASD: autism spectrum disorder; TD: typically developing youth.

*Within-group analyses.* Across lobules, the sensorimotor cerebellum showed functional connectivity with sensorimotor and visual regions, although the specific regions and extent of connectivity varied depending on the seed region (see **Figure 1** and **Table 2**). More specifically, all lobules showed extensive functional connectivity within the cerebellum (including with the vermis), and with the sensorimotor cortex, primary visual regions (i.e., intracalcarine and supracalcarine cortices), fusiform cortex, lingual gyrus, precuneus and cuneal cortex, and the brainstem.

**Table 2:** Cluster peak coordinates.

Lobule	Contrast	Region^1^	Coverage Area^2^	Voxels	Z-max	x	y	z
WITHIN-GROUP ANALYSES								
**Lob I-IV**	ASD	**Right lobules I-IV**	**Cerebellum:** [bilateral] lobules V, VI, Crus I, Crus II, VIIb, VIIIa, VIIIb, and IX, and vermis (VI, Crus II, VIIb, VIIIb, IX, X). **Cerebrum:** [bilateral] anterior and posterior parahippocampal gyrus, fusiform cortex, intracalcarine cortex, supracalcarine cortex, occipital pole, cuneal cortex. **Subcortical:** brainstem, bilateral hippocampus and right thalamus.	13239	10.4	14	-40	-18
		*Left lobules I-IV*			10.2	0	-48	-10
		*[Left] Lobules I-IV/lobule V/precuneus*			8.39	-6	-54	-4
		*[Left] Lingual Gyrus/precuneus*			6.55	-10	-54	2
		*Right precuneus*			6.3	18	-56	14
		*Vermis VIIIa*			5.89	2	-66	-34
		*[Right] Posterior cingulate gyrus/occipital gyri*			5.63	14	-48	2
		**Left precentral gyrus**	Left postcentral gyrus	982	5.07	-16	-28	72
		*[Right] Precentral gyrus/white matter/paracentral lobule*			4.7	6	-28	64
		*Right postcentral gyrus*			4.13	12	-36	68
**Lob V-VI**	ASD	**Left lobule VI/fusiform gyrus**	**Cerebellum:** [bilateral] lobules I-IV, V, Crus I, Crus II, VIIb, VIIIa, VIIIb, IX and X; and vermis (VI, Crus II, VIIb, VIIIa, VIIIb, IX and X). **Cerebrum:** [bilateral] posterior parahippocampal gyrus, posterior cingulate gyrus, fusiform cortex, intracalcarine cortex, supracalcarine cortex, occipital pole, cuneal cortex, precuneus, lingual gyrus, inferior and superior lateral occipital cortex, inferior temporal gyrus (temporooccipital and posterior), right middle temporal gyrus (temporooccipital), superior parietal lobule. **Subcortical:** brainstem, right hippocampus/thalamus	44568	11.5	-24	-58	-22
		*Right lobule VI/fusiform gyrus*			10.7	20	-66	-18
		*Right lobule V*			9.46	8	-62	-18
		*Left occipital fusiform gyrus*			9.28	-34	-78	-14
		*Left lingual gyrus*			9.12	-8	-66	-8
		**Left precentral gyrus**	–	1159	5.36	-44	-16	42
		*Left postcentral gyrus*			5.01	-52	-18	48
		*[Left] White matter/anterior supramarginal gyrus/parietal operculum*			4.11	-46	-30	34
		**[Right] Postcentral gyrus/precentral gyrus**	–	716	5.11	46	-18	42
		*White matter/right precentral gyrus/middle frontal gyrus*			3.57	30	-10	48
		**Left supplementary motor cortex**	–	588	5.95	0	-6	58
		*[Right] Supplementary motor cortex/superior frontal gyrus*			4.29	4	-4	74
		*Right anterior cingulate gyrus*			4.14	10	6	40
		**Left central opercular cortex**	[Left] Planum temporale, Heschl’s gyrus	280	5.02	-46	-20	18
		*[Left] Central opercular cortex/precentral gyrus*			4.56	-46	-10	12
		*Left parietal operculum cortex*			4.18	-34	-30	20
		*White matter*			3.62	-36	-40	18
		*Left insular cortex*			3.6	-34	-20	20
		**[Right] Superior parietal lobule/postcentral gyrus**	–	154	5.13	28	-40	52
**Lob VIII**	ASD	**[Right] Lobule VIIIa/VIIIb**	**Cerebellum:** [bilateral] lobules I-IV, V, VI, Crus I, Crus II, VIIb, IX and X; and vermis (VI, Crus II, VIIb, VIIIa, VIIIb and IX). **Cerebrum:** [bilateral] lingual gyrus, occipital fusiform gyrus, inferior and superior lateral occipital cortex, intracalcarine cortex, left supracalcarine cortex, occipital pole, cuneal cortex, middle temporal gyrus (temporooccipital), right inferior temporal gyrus (temporooccipital). **Subcortical:** brainstem	26379	10.1	26	-54	-56
		*Left Lobule VIIIa*			9.81	-32	-40	-50
		*Left Lobule VIIIb*			9.68	-18	-54	-54
		**[Right] Superior parietal lobule/postcentral gyrus**	Right precuneus	1684	5.97	28	-42	70
		*[Right] Superior lateral occipital cortex/superior parietal lobule*			5.52	12	-56	70
		*White matter*			3.84	20	-58	44
		**Left superior parietal lobule**	Left postcentral gyrus	1429	6.1	-20	-52	70
		*Left superior lateral occipital cortex*			4.56	-8	-62	64
		*Left precuneus*			4.56	-8	-54	66
		**[Right] Parietal operculum cortex/superior temporal gyrus**	Right planum temporale	864	5.71	58	-28	20
		*White matter/right anterior supramarginal gyrus/parietal operculum*			5.18	54	-32	34
		*Right anterior supramarginal gyrus*			4.02	68	-24	26
		**Left parietal operculum cortex**	–	592	4.79	-58	-30	18
		*[Left] Parietal operculum cortex/planum temporale*			4.63	-64	-32	26
		*[Left] Postcentral gyrus/anterior supramarginal gyrus*			3.52	-46	-28	36
		*[Left] Central opercular cortex/parietal operculum*			3.4	-46	-20	16
		**[Left] Supplementary Motor Cortex/paracentral lobule**	–	374	4.79	-2	-10	60
		*Left precentral gyrus*			4.47	-6	-14	70
		*[Right] Precentral Gyrus/superior frontal gyrus*			3.94	20	-14	70
		**Left precentral gyrus**	Left insular cortex	194	4.92	-62	0	6
		*Left central opercular cortex*			3.57	-46	-4	4
		*[Left] Planum polare/central opercular cortex*			3.53	-52	0	0
**Lob I-IV**	TD	**Left lobules I-IV**	**Cerebellum:** [bilateral] lobules V, VI, Crus I (left Crus I/left occipital fusiform cortex), Crus II, VIIb, VIIIa, VIIIb, IX, and right lobule X;, and vermis (VI, Crus II, VIIb, VIIIa, VIIIb and IX). **Cerebrum:** [bilateral] anterior and posterior parahippocampal gyrus, fusiform cortex, intracalcarine cortex, supracalcarine cortex, cuneal cortex, precuneus, posterior cingulate gyrus, lingual gyrus; [left] posterior inferior temporal gyrus and temporal pole. **Subcortical:** bilateral hippocampus, left amygdala, brainstem	15077	9.79	-2	-48	-20
		*Right lobules I-IV*			9.76	2	-48	-2
		*Vermis VIIIa/VIIb*			5.63	6	-68	-34
		**[Right] Precentral gyrus/paracentral lobule**	Right superior parietal lobule	1328	4.97	4	-30	70
		*Left postcentral gyrus*			4.89	-12	-36	70
		*Left precentral gyrus*			4.85	-2	-30	70
		*Right postcentral gyrus*			4.46	10	-38	76
		**Right parietal operculum cortex**	[Right] Heschl’s gyrus/insular cortex	130	3.96	40	-22	20
		*Right planum temporale*			3.59	38	-30	14
**Lob V-VI**	TD	**Left lobule VI/occipital cortex**	**Cerebellum:** [bilateral] lobules I-IV, V, Crus I, Crus II, VIIb, VIIIa, VIIIb, IX, and X; and vermis (VI, Crus II, VIIb, VIIIa, VIIIb and IX). **Cerebrum:** [bilateral] posterior parahippocampal gyrus, posterior cingulate gyrus, intracalcarine cortex, supracalcarine cortex, occipital pole, superior and inferior lateral occipital cortex, fusiform cortex, lingual gyrus, cuneal cortex, precuneus, middle temporal gyrus (temporooccipital), inferior temporal gyrus (temporooccipital and posterior). **Subcortical:** brainstem, right hippocampus	44566	10.9	-10	-70	-16
		*Left lobule VI/fusiform cortex*			9.99	-28	-62	-18
		*Right lobule VI/occipital cortex*			9.8	14	-72	-16
		*Vermis VI*			9.73	6	-74	-20
		*Left occipital fusiform gyrus*			9.28	-36	-68	-18
		*Right lingual Gyrus*			9.21	18	-64	-12
		**Right postcentral gyrus**	–	552	4.82	32	-34	70
		*[Right] Superior lateral occipital cortex/superior parietal lobule*			4.17	28	-58	60
		**Right precentral gyrus**	–	496	5.42	44	-12	50
		Right *Postcentral gyrus*			4.19	42	-20	42
		*[Right] Anterior supramarginal gyrus/postcentral gyrus*			3.29	42	-34	46
		**Left superior parietal lobule**	Left postcentral gyrus	334	4.56	-30	-46	62
		*Left superior lateral occipital cortex*			3.89	-20	-60	62
		**Left parietal Operculum Cortex**	Left anterior supramarginal gyrus	159	4.18	-46	-38	24
		*[Left] Parietal Operculum Cortex/superior temporal gyrus*			3.21	-60	-28	20
		**Right parietal operculum cortex**	Right anterior supramarginal gyrus	139	5.19	56	-22	20
		*[Right] Postcentral gyrus/central opercular cortex*			3.58	62	-12	16
**Lob VIII**	TD	**Right lobule VIIIb**	**Cerebellum:** [bilateral] lobules I-IV, V, VI, Crus I, Crus II, VIIb, IX and X; and vermis (VI, Crus II, VIIb, VIIIa, VIIIb and IX). **Cerebrum:** [bilateral] postcentral gyrus, superior parietal lobule, left posterior parahippocampal gyrus. parietal operculum cortex, planum temporale, right anterior and posterior supramarginal gyrus, lingual gyrus, fusiform cortex, intracalcarine cortex, supracalcarine cortex, occipital pole, inferior and superior lateral occipital cortex, cuneal cortex, precuneus, middle temporal gyrus (temporooccipital), inferior temporal gyrus (temporooccipital and left posterior). **Subcortical:** brainstem	39782	10.3	22	-48	-54
		*Right lobule VIIIa*			9.9	32	-46	-52
		*Left lobule VIIIb*			9.38	-22	-48	-52
		*Left lobule VIIIa*			9.24	-24	-60	-54
		**[Left] Anterior supramarginal cortex/parietal operculum cortex**	[Left] Postcentral gyrus, central opercular cortex	667	5.25	-58	-32	26
		**[Left] Superior parietal lobule/postcentral gyrus/white matter**		162	4.49	-32	-40	44
BETWEEN-GROUP ANALYSES								
**Lob I-IV**	ASD>TD	[Right] Hippocampus/thalamus (lateral pulvinar nucleus)	Right posterior parahippocampal gyrus, right lingual gyrus, brainstem; cerebellar [bilateral] lobules I-IV, left lobules V and IX, vermis IX and X	542	3.6	20	-32	-6
	ASD>TD	White matter	–	20	2.78	34	-34	32
**Lob V-VI**	ASD>TD	White matter/right superior lateral occipital cortex	–	17	2.6	26	-64	32
**Lob V-VI**	ASD<TD	Left crus II	Right crus II; bilateral crus I; right lobule VI; vermis VI and crus II	576	4.2	0	-82	-34
**Lob VIII**	ASD<TD	Right crus I	[Right] Cerebellar lobule VI, occipital fusiform gyrus, lingual gyrus, temporal occipital fusiform cortex	448	3.41	52	-66	-28
SOR ANALYSES								
**Lob I-IV**	SORneg	Supplementary motor cortex/bilateral superior frontal gyrus	–	427	4.47	6	-4	66
**Lob V-VI**	SORneg	Right crus I	Right crus II	653	4.04	42	-66	-38
**Lob VIII**	SORneg	White matter	Lateral and medial prefrontal cortex	433	4	18	30	-2
	SORpos	Left precentral gyrus	[Left] Postcentral gyrus, superior parietal lobule, precuneus	910	4.03	-14	-30	68
	SORpos	White matter/precuneus/paracentral lobule	[Left] Superior parietal lobule, superior lateral occipital cortex	708	4.38	-18	-42	36

^1^Regions listed in bold are peaks; those listed in italics are subpeaks within the same cluster as the coordinates above them.

^2^Additional regions covered by the cluster beyond the peak are described.

x, y, and z refer to the left–right, anterior–posterior, and inferior–superior dimensions, respectively; Z refers to the Z-score at those coordinates (local maxima or submaxima). Voxels indicate cluster size. Within- and between-group analyses are cluster corrected for multiple comparisons, z>3.1 (within-group) or z>2.3 (between-group and correlations with SOR), p<.05. ASD>TD and ASD<TD contrasts were masked by the ASD and TD within-group contrast, respectively, to display clusters that show greater positive connectivity in ASD compared to TD, and vice versa. ASD, autism spectrum disorder; TD, typically developing youth; SOR, sensory over-responsivity.

In both ASD and TD groups, lobules I-IV were additionally functionally connected with posterior and anterior parahippocampal gyrus, posterior cingulate gyrus and bilateral hippocampus (**Figure 1A**, left; **Table 2**). Lobules V-VI showed additional connectivity with the posterior parahippocampal gyrus, posterior cingulate gyrus, lateral occipital cortex, occipital pole, temporal cortex, higher-order sensory regions (i.e., superior parietal lobule, anterior supramarginal gyrus and operculum), and right hippocampus. Lobule VIII showed further functional connectivity with the lateral occipital cortex, occipital pole, higher-order sensory regions (i.e., superior parietal lobule, anterior supramarginal gyrus, operculum and planum temporale) and temporal cortex.

*Between-group analyses.* In representing between-group analyses (**Figure 1**), ASD>TD contrasts were masked posthoc with the within-group contrast representing positive functional connectivity in ASD at z>2.3 to display clusters that show greater positive connectivity in ASD compared to TD. Similarly, ASD<TD contrasts were masked by the within-group contrast demonstrating positive functional connectivity in TD at z>2.3 to display clusters that show greater positive connectivity in TD compared to ASD.

The ASD and TD groups showed significant differences in connectivity across cerebellar seeds. Lobules I-IV showed stronger connectivity in ASD compared to TD with right hippocampus/thalamus (lateral pulvinar nucleus), right posterior parahippocampal gyrus, right lingual gyrus and the brainstem as well as with cerebellar lobules I-IV, left lobules V and IX, and vermis IX and X (**Figure 1A**, right; **Table 2**). In ASD, lobules V-VI showed stronger connectivity compared to the TD group with right superior lateral occipital cortex/white matter, and weaker connectivity with cerebellar bilateral crus II, bilateral crus I, right lobule VI, and vermis VI and crus II (**Figure 1B**, right; **Table 2**). The ASD group showed weaker lobule VIII functional connectivity with right crus I, right lobule VI, fusiform cortex, and lingual gyrus compared to the TD group.

### 3.3 SOR correlations with cerebellar connectivity

To determine whether sensorimotor cerebellar connectivity was associated with SOR in ASD, we used SOR as a regressor in whole-brain analyses, co-varying for anxiety.

**Figure 2:**
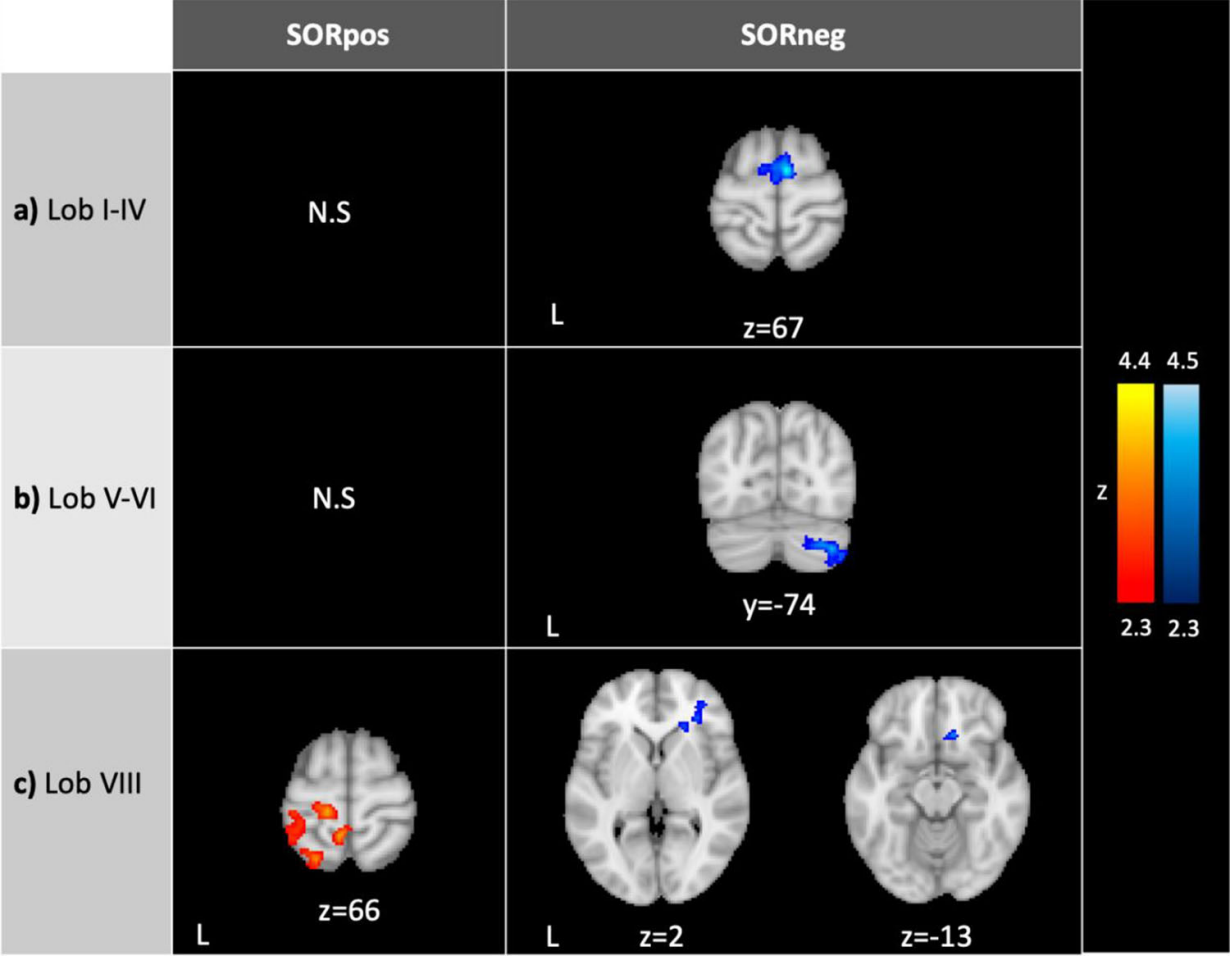
Connectivity of the sensorimotor cerebellum in ASD correlating with SOR. SORpos: Regions where resting-state functional connectivity with lobules I-IV, lobules V-VI and lobule VIII correlates positively with SOR severity (*left*, in red). SORneg: Regions where resting-state functional connectivity with the cerebellar lobules correlates negatively with SOR (*right*, in blue). There were no significant clusters where connectivity with lobules I-IV and V-VI correlated positively with SOR. SOR was entered as a bottom-up regressor in analyses, and anxiety was included as a regressor of no-interest. In right c), the images show the same cluster. Contrasts were thresholded at z>2.3 and cluster corrected at *p*<0.05. SOR, sensory over-responsivity; N.S., no significant clusters.

SOR severity correlated negatively with lobule I-IV connectivity with supplementary motor cortex/superior frontal gyrus (**Figure 2**). ASD youth with higher SOR had weaker lobule V-VI connectivity with cerebellar right Crus I and II (**Table 2**). SOR was negatively correlated also with connectivity between lobule VIII and lateral and medial prefrontal cortex (lPFC and mPFC, respectively); and positively correlated with connectivity between lobule VIII and precentral and postcentral gyri, superior parietal lobule, superior lateral occipital cortex, paracentral lobule, and precuneus.

**Figure 3:**
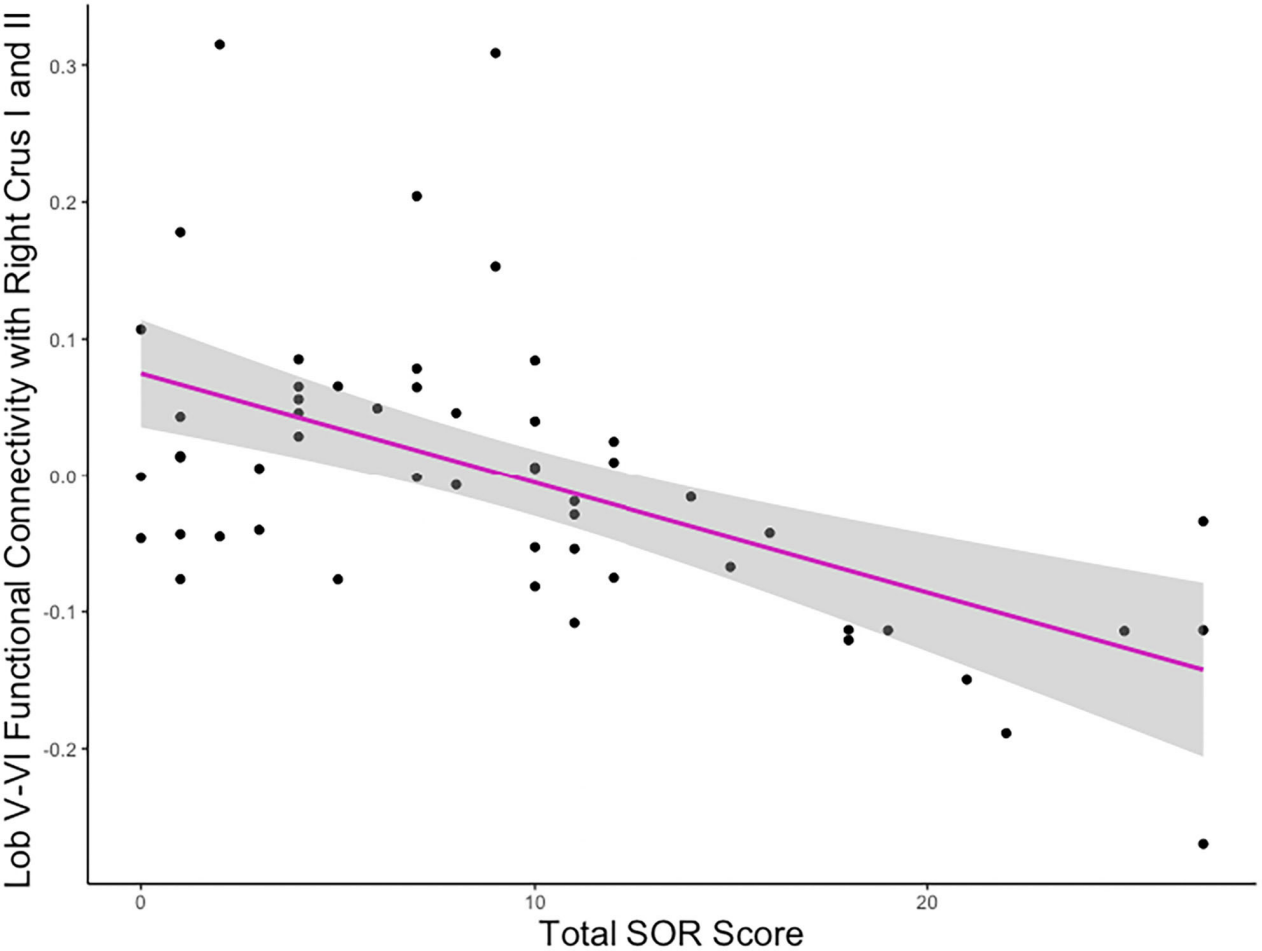
Association between lobule V-VI connectivity strength and SOR. Example plot demonstrating the negative correlation between SOR severity and the strength of connectivity between lobules V-VI and the cluster covering right crus I and II, also portrayed in **Figure 2B** (right). Y values are residuals after partialling out the effect of anxiety, which was covaried in the analyses.

To ensure that correlations with SOR (e.g., **Figure 3**) were not driven by outliers, we extracted parameter estimates from regions where connectivity strength was significantly correlated with SOR. We evaluated the parameter estimates for outliers (i.e., ± 3 interquartile range) and detected no outliers. We additionally visually inspected parameter estimate – SOR severity scatterplots for any potential outliers. All the correlations remained significant after removing any potential connectivity-SOR outliers, indicating that these correlations were not driven by outliers.

We additionally investigated the effect of anxiety on cerebellar functional connectivity over and above SOR severity, and found no significant associations between anxiety and cerebellar connectivity.

We replicated all our analyses controlling for age to account for age variability in our sample and found comparable results with age as a covariate (**Supplementary Figures 4, 5**).

## 4 Discussion

The focus of this study was to investigate resting-state functional connectivity of the sensorimotor cerebellum in ASD compared to TD youth. Additionally, we examined the association between cerebellar connectivity and sensory over-responsivity within ASD youth. The sensorimotor cerebellum overall showed widespread connections within the cerebellum and with sensorimotor and visual areas, brainstem, precuneus and cuneus. Each of the three seeds also displayed distinct areas of connectivity with the limbic system (lobules I-IV and lobules V-VI) and higher-order sensory regions (lobules V-VI and lobule VIII). These connectivity patterns were, for the most part, consistent across ASD and TD participants. However, the ASD participants did show some significant connectivity differences, particularly within the cerebellum, and between the sensorimotor cerebellum and the hippocampus, thalamus, visual regions, and the brainstem. In addition, within youth with ASD, SOR was associated with sensorimotor cerebellar connectivity with both sensorimotor and visual regions and regions implicated in cognitive and socio-emotional processing. To our knowledge, this is the first study that examined cerebellar atypicalities in the context of sensory over-responsivity in autism.

### 4.1 Functional connectivity of the sensorimotor cerebellum

For the purposes of the current study, we delineated the sensorimotor cerebellum as three seeds involving lobules I-IV, lobules V-VI and lobule VIII, due to the extensive literature indicating their involvement in sensorimotor processing (e.g., 9, 11, 15, 34). Our results indicated that for both the ASD and TD groups, all three seeds (i.e., lobules I-IV, V-VI and VIII) showed connectivity within the cerebellum, including within the lobule in consistence with Bernard et al. (53), and widespread connectivity with sensorimotor regions, especially visual, somatosensory and motor cortices. Thus, in contrast to prior studies (36), we found connectivity with visual regions across all examined lobules, although lobules V-VI and lobule VIII showed more extensive functional connectivity with the occipital lobe, including the lateral occipital cortex and occipital pole. In terms of sensorimotor cortex, lobules I-IV were also predominantly functionally connected with primary sensory and motor cortices, whereas lobules V-VI and VIII showed more extensive connectivity with higher-order sensory regions. Taken together, our results suggest that processing of sensory signals is integrated across multiple cerebellar lobules, though certain lobules may play a more distinct role in higher-order processing of sensory information.

In addition to connectivity with sensory and motor cortical regions, the sensorimotor cerebellum was functionally connected with limbic regions. We found functional connectivity between the limbic system (i.e., hippocampus, parahippocampal gyrus and posterior cingulate gyrus) and lobules I-IV and V-VI across both ASD and TD participants. These findings are consistent with the growing literature that reports functional connectivity between the cerebellum and the hippocampus (see 54 for a review). While the type of information encoded in this circuitry cannot be determined from the current resting-state study, cerebellar-limbic connectivity may reflect that sensory processing is interrelated with cognitive and emotional processing. Additionally, these results demonstrating connectivity between limbic regions and cerebellar lobules associated with sensory processing also challenge the idea that cerebellar subregions can be divided into specialized sensorimotor and supramodal cognitive zones as initially proposed (11, 36, 55). These findings are thus also consistent with research showing that sensorimotor cerebellar lobules are activated during cognitive tasks (56, 57) and contain representations of non-sensorimotor cerebral networks (e.g., 32, 55).

### 4.2 Diagnostic group differences in sensorimotor cerebellum connectivity

While the cerebellar networks identified here were overall consistent across ASD and TD groups, there were some notable regions of diagnostic group differences, particularly with sensory regions and hippocampus as well as within the cerebellum.

Visual networks showed distinct patterns of cerebellar connectivity in ASD compared to TD. We found enhanced connectivity between lobules I-IV and lingual gyrus and between lobules V-VI and lateral occipital cortex, while lobule VIII displayed weaker connectivity with lingual gyrus and the fusiform cortex in ASD. Differences in cerebellar connectivity with these visual regions involved in functions such as object recognition and face processing might contribute to atypical processing of visual information in ASD (e.g., 58–60). Furthermore, the sensorimotor cerebellum, particularly lobules I-IV, displayed stronger functional connectivity with the right thalamus in ASD. The thalamus plays an important role in relaying and integrating sensory information (61) and connects cerebello-cortical loops (62). Critically, thalamocortical networks show distinct connectivity patterns in autism (63–67), and thalamic connectivity has been implicated in sensory challenges in ASD youth (26, 68) and in infants with high familial likelihood for ASD (69). Greater connectivity between thalamus and sensorimotor cerebellum therefore may be another indicator of increased processing of sensory information and atypical sensory error signals (see discussion of prediction models below).

Additionally, we found stronger connectivity in ASD between the sensorimotor cerebellum (i.e., lobule I-IV) and the hippocampus compared to in TD youth. Along with the cerebellum, the hippocampus shows structural and functional differences in ASD (70–73), and is also implicated in autistic features, including challenges in social behavior and memory processing as well as strengths in visuo-spatial tasks (see 74 for a review). Importantly, recent literature on hippocampal function suggests that the hippocampus is involved not only in spatial memory, but also in the organization of different kinds of information (i.e., cognitive mapping (74, 75); to help us adapt to changes in our environment. Greater connectivity between the hippocampus and the sensorimotor cerebellum could thus be related to greater neural resources devoted to the organizational mapping of sensory information in ASD, which might contribute to both sensory strengths and challenges seen in ASD. This idea is consistent with previous findings demonstrating stronger reactivity in response sensory stimulation in both the hippocampus and cerebellum in autistic youth (24).

Finally, compared to TD, the ASD group showed greater within-cerebellum connectivity of lobules I-IV, and weaker within-cerebellum connectivity of lobules V-VI and VIII, including with the cerebellar crus I and II. These results are in alignment with previous research showing differences in local functional connectivity of the cerebellum in autism (76, 77), and may indicate distinct functional organization of the cerebellum in ASD. Particularly, a decreased connectivity between the sensorimotor cerebellum and crus I and II, which are functionally connected with the PFC, may indicate reduced integration of processing across functionally distinct cerebellar networks (78). More research is needed to determine how such organizational differences might lead to differences in information processing.

### 4.3 Sensorimotor cerebellum connectivity and SOR

Given the role of the cerebellum in sensorimotor processing and its demonstrated functional connectivity differences with sensory processing regions in ASD, we further examined whether sensorimotor cerebellar connectivity was associated with SOR symptoms in ASD. We found that more severe SOR symptoms were related to heightened cerebellar connectivity with the primary motor cortex and primary and higher-order sensory regions in ASD, providing a link between atypical cerebellar function in ASD and SOR. This finding is in alignment with Oldehinkel et al. (29) who found stronger cerebellum-sensorimotor network connectivity to be associated with more severe sensory symptoms. Previous research has shown that more severe SOR is associated with reduced habituation in sensory regions (25), suggesting that individuals with ASD and SOR might not adapt to sensory stimuli in the environment. The cerebellum is involved in error-based learning and maintains prediction models of sensory consequences of actions (79). Atypicalities in cerebellar connectivity with the sensorimotor network could be a sign of altered cerebellar signaling of sensory predictions to the sensorimotor network in ASD. This alteration in signaling could indicate lowered predictability of sensorimotor signals and consequently of the external world in ASD (5, 80), leading to reduced habituation and heightened reaction to sensory information as seen in SOR. In addition to alterations in habituation, more severe SOR has previously been linked to lower PFC-amygdala connectivity during sensory stimulation (24, 25), indicating that top-down emotion regulation through the PFC may be reduced in ASD youth with severe SOR. In the current study, more severe SOR in ASD was also associated with weaker sensorimotor cerebellum-PFC connectivity, such that youth with *higher* SOR showed *lower* lobule VIII connectivity with mPFC and lPFC. Moreover, more severe SOR was additionally associated with weaker functional connectivity between the sensorimotor cerebellum and crus I and II, the part of the cerebellum that is particularly implicated in socio-emotional function (34, 81, 82). Taken together, these findings suggest that reduced sensorimotor cerebellum connectivity with supramodal regions – important to cognition and emotional processing – as well as increased connectivity with sensorimotor cortex, may contribute to SOR experiences in ASD, and are in line with research demonstrating heightened allocation of mental resources to sensory information, sometimes at the expense of processing other types of information, in autism (e.g., 83, 84).

### 4.4 Limitations and future directions

The current study has multiple strengths, including examining shared and distinct connectivity of three different sensorimotor cerebellar regions. We also investigated for the first time the role of the cerebellum in SOR in ASD youth, with a relatively large sample compared to prior studies. Nevertheless, our investigation has some limitations. First, while there are many dimensions of sensorimotor function such as sensory acquisition, discrimination and integration, the current study focused on sensory modulation (i.e., how to regulate and respond to incoming sensory information), and specifically SOR, in ASD. Previous research in neurotypical populations examined the role of the cerebellum in other aspects of sensory processing (85), such as in sensory discrimination and acquisition (86, 87) as well as with tasks that involve biological motion (88) and pain stimuli (89). Moreover, atypicalities in multiple aspects of sensorimotor processing have been reported in autism, including differences in integration of sensory feedback (e.g., 90, 91), altered activation and connectivity during motor tasks (e.g., 12) and atypical updating of sensory prediction models (e.g., 92). While SOR warrants particular attention from researchers due to its impairing nature and its developmental progression across adolescence in ASD, future research should characterize the involvement of the cerebellum in the full range of sensory processing atypicalities seen in ASD, including other sensory modulation atypicalities (i.e., sensory under-responsivity and sensory seeking).

Second, in the current study, we assessed SOR severity with parent-report. While parent-report (along with self-report) is one of the most common methods to assess sensory features in ASD samples (93, 94), it may not capture all aspects of one’s sensory experiences. In fact, parent-reported, self-reported and observed sensory assessments may tap into different aspects of sensory processing and complement one another (42, 95). For example, while parent-report might involve insights into behavioral responses to sensory stimuli and past history of sensory challenges, self-report could better represent internal experience, especially in older participants, and observed assessments (in the presence of an experimenter) may capture ability to regulate sensory responses rather than purely sensory experience (42, 95–97). In investigating the link between SOR and cerebellar function, future studies could utilize an integrated (i.e., self-reported, parent-reported and observed SOR) approach to measure SOR comprehensively and also interrogate how cerebellar connectivity relates to different aspects of sensory processing.

Third, while our sample included a relatively large number of females compared to many autism studies, the current study was underpowered to examine sex differences in cerebellar connectivity and its relationship with SOR. Emerging evidence shows that that sensory symptoms and restricted and repetitive behaviors (RRBs) may have different underlying neurobiology in girls versus boys with ASD (e.g., 27, 98), and thus future studies should examine sex differences in cerebellar connectivity and their differential relationship to SOR. Fourth, the sample in the current study is a pediatric sample including youth in middle childhood and adolescence. The development of the cerebellum continues through adolescence, with different parts of the cerebellum reaching their mature state at distinct times during development (99–101). Cerebellar responses to aversive sensory information may also change across development (43). Thus, future studies should investigate how connectivity of the sensorimotor cerebellum evolves during development, especially during adolescence, and how these developmental changes relate to SOR severity. Fifth, in the current study, the sensorimotor regions within the cerebellum were defined based on the neuroanatomy of the brain region. However, recent research has shown that anatomical cerebellar regions do not map on to functional subdivisions (56), indicating that the sensorimotor cerebellum might be more accurately defined by functional masks. In fact, sensorimotor cerebellar lobules encompass functional regions that are activated during non-sensorimotor processes, such as theory of mind, working memory and verb generation tasks (56, 57). Lobule VI, in particular, is involved in cognition and executive function (102). Thus, anatomical sensorimotor seeds might include non-sensorimotor subsections of the cerebellum as well as exclude sensorimotor functional regions outside of the anatomically defined lobules. Hence, future studies should use functionally defined sensorimotor cerebellum masks to assess the connectivity of the sensorimotor regions within the cerebellum. Furthermore, while we conducted analyses with cerebellar seeds, alternative analytic approaches to examine cerebellar connectivity (e.g., investigating cerebellar connectivity with cerebral seeds or with smaller seed regions) could be explored in future studies. Finally, while SOR is particularly prevalent in the autistic population, TD individuals also experience SOR symptoms. In fact, our original sample included one TD participant with elevated SOR severity, who was excluded from our final sample. Future research should also investigate SOR variability in other populations and whether altered cerebellar functional connectivity is involved in neural mechanisms of SOR in typical development or other non-autistic populations.

## 5 Conclusion

In the current study, we found that, across both diagnostic groups, the three sensorimotor cerebellar seeds that were examined, namely lobules I-IV, V-VI and VIII, were all functionally connected with sensorimotor and visual areas, brainstem, precuneus and cuneus, but showed distinctions in their connectivity with limbic and higher-order sensory regions. Youth with ASD had atypical connectivity of the cerebellum with the thalamus, hippocampus, parahippocampal gyrus, brainstem, and the visual cortex as well as within the cerebellum. In relation to SOR, we showed that more severe SOR is associated with *stronger* connectivity between the sensorimotor cerebellum and cerebral sensorimotor regions and precuneus, and *weaker* connectivity between the cerebellum and cognitive and socio-emotional regions, particularly the prefrontal cortex. These findings provide evidence for a link between functional cerebellar atypicalities in ASD and SOR for the first time. The current study adds to the recent literature indicating the involvement of cerebellar atypicalities in the differences in perception and behavior seen in ASD. Taken together with past research that described a role for the cerebellum in socio-communicative symptoms of ASD, our findings suggest that the cerebellum should be considered in the study of ASD and as well as the study of atypical sensory processing in other populations.

## Data availability statement

The raw data supporting the conclusions of this article will be made available by the authors, without undue reservation.

## Ethics statement

The studies involving humans were approved by University of California, Los Angeles, Institutional Review Board. The studies were conducted in accordance with the local legislation and institutional requirements. Written informed consent for participation in this study was provided by the participants’ legal guardians/next of kin.

## Author contributions

MC: Writing – review & editing, Writing – original draft, Visualization, Formal analysis, Data curation, Conceptualization. NO: Writing – review & editing, Methodology, Investigation, Data curation. KC: Writing – review & editing, Investigation, Data curation. JJ: Writing – review & editing, Investigation, Data curation. SB: Writing – review & editing, Supervision, Methodology, Funding acquisition, Conceptualization. MD: Writing – review & editing, Supervision, Resources, Methodology. SG: Writing – review & editing, Supervision, Resources, Project administration, Methodology, Funding acquisition, Conceptualization.

## Footnotes

1
www.fmrib.ox.ac.uk/fsl
