# Supplementary material for: Functional connectivity of the sensorimotor cerebellum in autism: associations with sensory over-responsivity
Source: Front Psychiatry. 2024 Mar 25;15:1337921. doi: 10.3389/fpsyt.2024.1337921 (PMC10999625; doi:10.3389/fpsyt.2024.1337921)
Supplement: Supplementary file 1 [file DataSheet_1.docx]

**SUPPLEMENTAL INFORMATION**

1. Cerebellar seeds


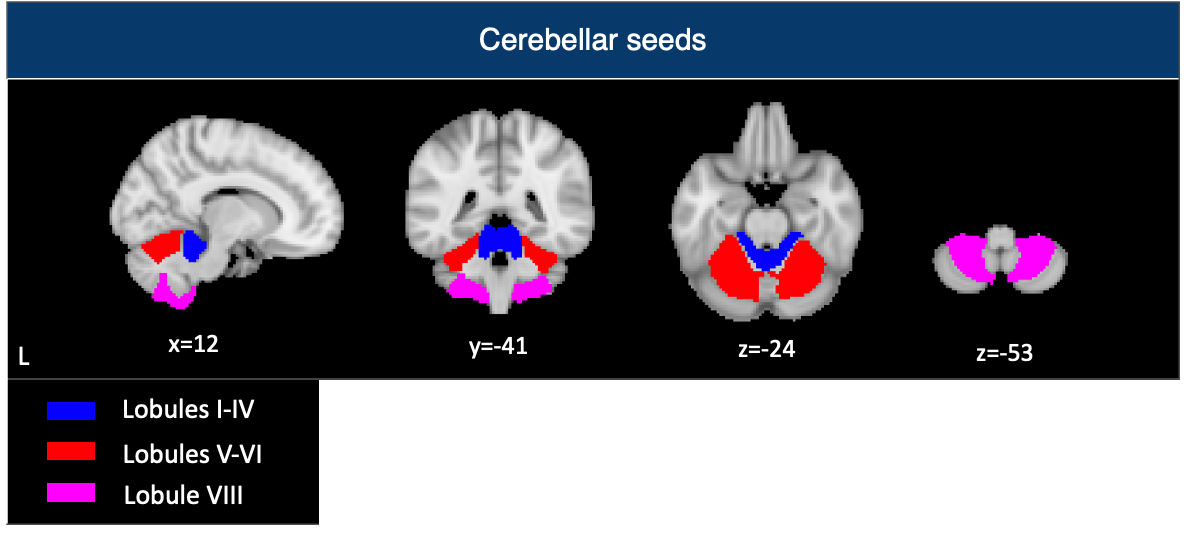


**Supplemental Figure 1. Anatomical cerebellar seeds.** Figure displaying cerebellar seeds used in functional connectivity analyses. All the seeds were thresholded at 75%, as described in the Methods section. Blue: lobules I-IV; red: lobules V-VI; pink: lobule VIII.

1. Analyses thresholded at z>2.7

Between-group and SOR correlation analyses were repeated at a more stringent threshold of z>2.7. Similar to Figure 1, between-group analyses were masked with within-group functional connectivity maps thresholded at z>2.3 (i.e., ASD<TD contrast thresholded at z>2.7 was masked with within-group TD positive connectivity results (at z>2.3) to display reduced positive connectivity in ASD compared to TD).


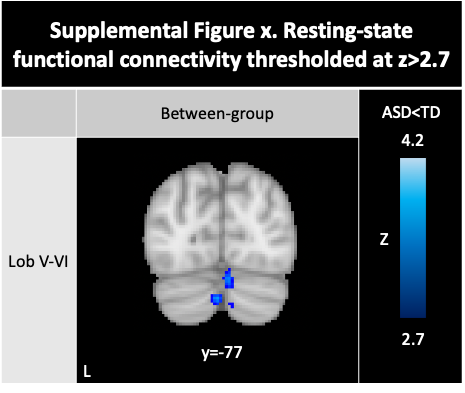


**Supplemental Figure 2. Between-group resting-state functional connectivity differences of the sensorimotor cerebellum at z>2.7.** Between-group functional contrasts were thresholded at z>2.7, cluster corrected at p<0.05. Full-scale IQ was included as covariates of no interest in between-group analyses. At z>2.7 threshold, only lobules V-VI showed differences in connectivity between ASD and TD groups (blue: ASD<TD). In parallel with Figure 1, ASD<TD results at z>2.7 were masked by the TD within-group contrast at z>2.3 to display clusters that show reduced positive connectivity in ASD compared to TD. ASD: autism spectrum disorder; TD: typically developing youth.


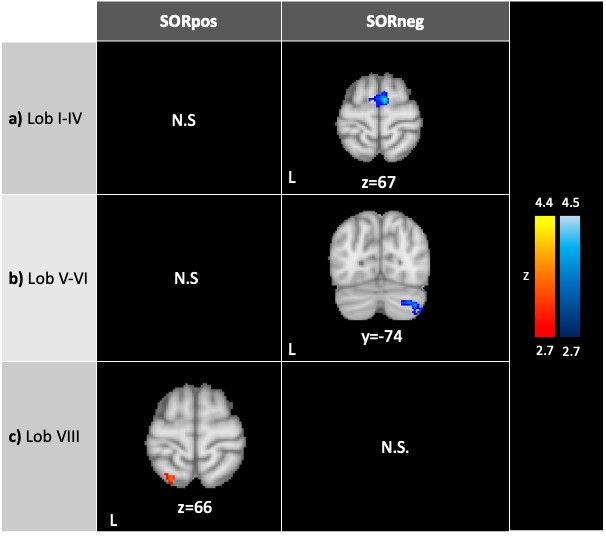


**Supplemental Figure 3. Connectivity of the sensorimotor cerebellum in ASD correlating with SOR, thresholded at z>2.7.** Regions where resting-state functional connectivity with lobules I-IV, lobules V-VI and lobule VIII positively (*left*, in red) and negatively (*right*, in blue) correlates with SOR. There were no significant clusters where connectivity with lobules I-IV and V-VI correlated positively and where lobule VIII correlated negatively with SOR. SOR was entered as a bottom-up regressor in analyses, and anxiety was included as a covariate of no interest. Contrasts were thresholded at z>2.7 and cluster corrected at p<0.05. SOR: sensory over-responsivity; N.S.: no significant clusters.

1. Analyses with anxiety as a covariate

To assess the effect of anxiety on cerebellar connectivity over and above the effect of SOR severity, we ran additional analyses with anxiety as a bottom-up regressor in the ASD group while covarying SOR severity.

We found no significant effect of anxiety on cerebellar connectivity while controlling for SOR severity.

1. Analyses with age as a covariate

Given the variability in age within our sample, we repeated all the analyses while controlling for age. We conducted within-group, between-group and SOR correlation analyses as described in the Methods section, with age as an added covariate of no interest.

We found the results of analyses with (Supplemental Figures 4 and 5) and without age as a covariate (Figures 1 and 2) to be highly consistent, except for between-group differences in lobule VIII connectivity (Supplemental Figure 4).


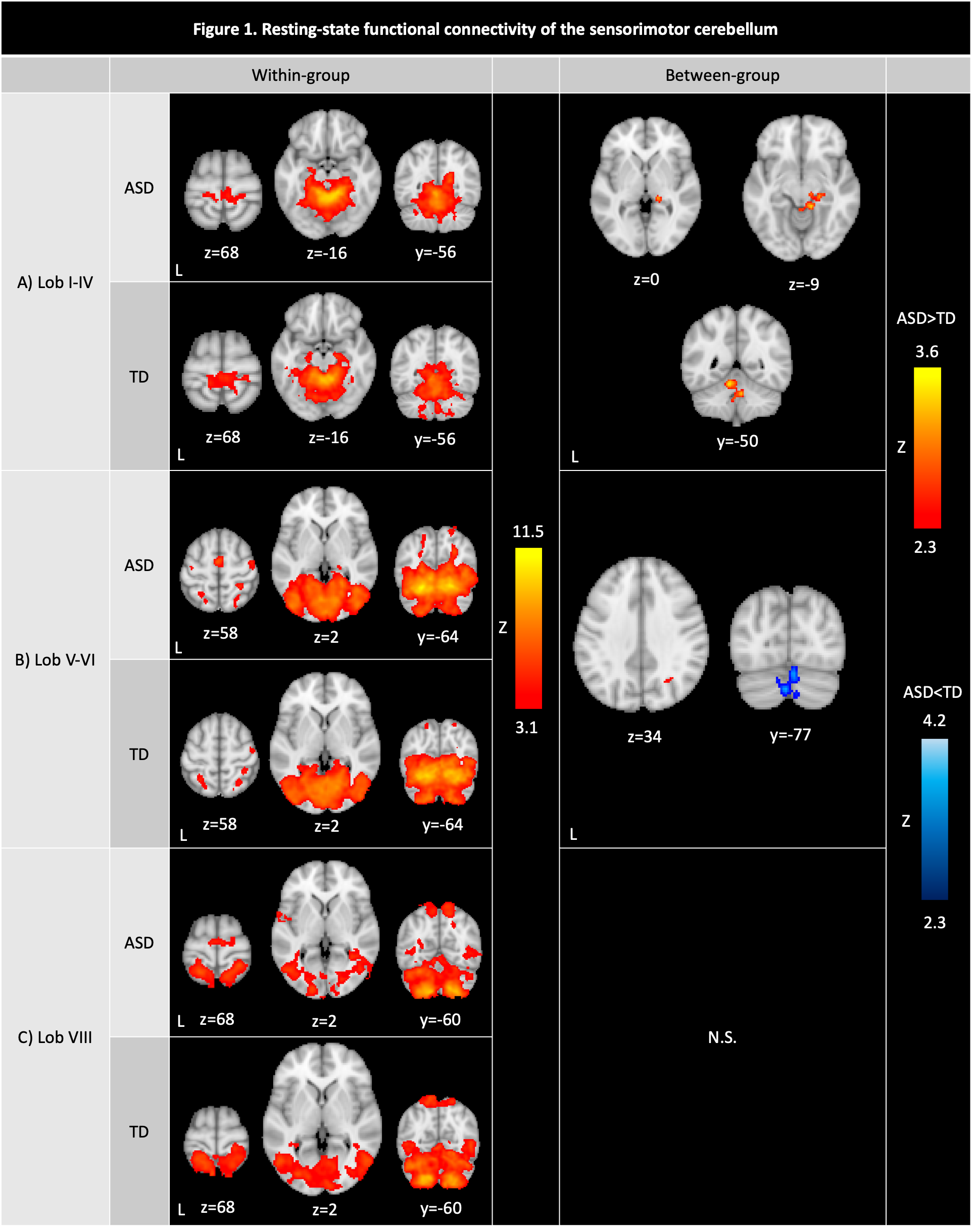


**Supplemental Figure 4. Whole-brain resting-state functional connectivity of the sensorimotor cerebellum, with age as a covariate of no interest.** (*left*) Within-group functional contrasts were thresholded at Z>3.1, cluster corrected at p<0.05. (*right*) Between-group functional contrasts were thresholded at Z>2.3, cluster corrected at p<0.05. Age was covaried in within-group analyses, and full-scale IQ and age were included as covariates of no interest in between-group analyses. Cerebellar lobules showed differences in connectivity between ASD and TD groups (red: ASD>TD; blue: ASD<TD). ASD>TD was masked by the ASD within-group contrast to display clusters that show greater positive connectivity in ASD compared to TD. Similarly, ASD<TD was masked by the TD within-group contrast to display clusters that show reduced positive connectivity in ASD compared to TD. ASD: autism spectrum disorder; TD: typically developing youth.

**Supplemental Figure 5. Connectivity of the sensorimotor cerebellum in ASD correlating with SOR, with age as a covariate of no interest.** Regions where resting-state functional connectivity with lobules I-IV, lobules V-VI and lobule VIII positively (*left*, in red) and negatively (*right*, in blue) correlates with SOR. There were no significant clusters where connectivity with lobules I-IV and V-VI correlated positively with SOR. SOR was entered as a bottom-up regressor in analyses, and anxiety and age were included as covariates of no interest. Note: In right c), the images show the same cluster. Contrasts were thresholded at z>2.3 and cluster corrected at p<0.05. SOR: sensory over-responsivity; N.S.: no significant clusters.
